# The epidemiology and medical management of low back pain during ambulatory medical care visits in the United States

**DOI:** 10.1186/1750-4732-2-11

**Published:** 2008-11-24

**Authors:** John C Licciardone

**Affiliations:** 1 The Osteopathic Research Center, University of North Texas Health Science Center, Fort Worth, TX, USA

## Abstract

**Background:**

Low back pain (LBP) is a common symptom.

**Methods:**

Patient visits attributed to LBP in the National Ambulatory Medical Care Survey (NAMCS) during 2003–2004 served as the basis for epidemiological analyses (n = 1539). The subset of patient visits in which LBP was the primary reason for seeking care (primary LBP patient visits) served as the basis for medical management analyses (n = 1042). National population estimates were derived using statistical weighting techniques.

**Results:**

There were 61.7 million (SE, 4.0 million) LBP patient visits and 42.4 million (SE, 3.1 million) primary LBP patient visits. Only 55% of LBP patient visits were provided by primary care physicians. Age, geographic region, chronicity of symptoms, injury, type of physician provider, and physician specialty were associated with LBP patient visits. Age, injury, primary care physician status, type of physician provider, and shared physician care were associated with chronicity of LBP care. Osteopathic physicians were more likely than allopathic physicians to provide medical care during LBP patient visits (odds ratio [OR], 2.61; 95% confidence interval [CI], 1.75–3.92) and chronic LBP patient visits (OR, 4.39; 95% CI, 2.47–7.80). Nonsteroidal anti-inflammatory drugs (NSAIDs) and narcotic analgesics were ordered during 14.2 million (SE, 1.2 million) and 10.5 million (SE, 1.1 million) primary LBP patient visits, respectively. Drugs (OR, 0.29; 95% CI, 0.13–0.62) and, specifically, NSAIDs (OR, 0.40; 95% CI, 0.25–0.64) were ordered less often during chronic LBP patient visits compared with acute LBP patient visits. Overall, osteopathic physicians were less likely than allopathic physicians to order NSAIDs for LBP (OR, 0.43; 95% CI, 0.24–0.76). Almost two million surgical procedures were ordered, scheduled, or performed during primary LBP patient visits.

**Conclusion:**

The percentage of LBP visits provided by primary care physicians in the United States remains suboptimal. Medical management of LBP, particularly chronic LBP, appears to over-utilize surgery relative to more conservative measures such as patient counseling, non-narcotic analgesics, and other drug therapies. Osteopathic physicians are more likely to provide LBP care, and less likely to use NSAIDs during such visits, than their allopathic counterparts. In general, LBP medical management does not appear to be in accord with evidence-based guidelines.

## Background

Back pain is a common symptom in industrialized nations that is responsible for substantial morbidity, impairment, and disability. Low back problems have been leading reasons for patient visits and health care costs despite measures to control access to services and contain costs [1]. Health care costs and productivity losses, most often associated with chronicity [2], may be in excess of $50 billion annually in the United States [3].

Back problems almost always consist of or co-exist with pain, including back-related leg pain or sciatica [4]. Although generically referred to as "back problems"[4] or "back pain," most cases involve the lower back. Low back pain (LBP) is defined as pain localized between the twelfth rib and the inferior gluteal folds, with or without leg pain [5]. Low back pain is often classified as acute when it lasts for less than 6 weeks, subacute when it lasts between 6 weeks and 3 months, and chronic when it persists for longer than 3 months [6]. The vast majority of LBP cases involve a non-specific etiology. Yellow flags (including individual, psychosocial, and occupational factors [7]) are prognostic factors for occurrence and chronicity of such non-specific LBP, whereas red flags are signs or symptoms that have come to be associated with specific pathological causes of LBP [5].

Historically, LBP has taken up a large part of primary care practice [8]. It has been the second leading cause of office visits to primary care physicians [9], and the most common reason for office visits to occupational medicine physicians, orthopedic surgeons, and neurosurgeons [4]. Allopathic family (general) medicine physicians, osteopathic physicians, chiropractors, orthopedic surgeons, and other specialists are the main providers of LBP care in the United States [10]. A variety of treatments for LBP have been introduced into clinical practice, including educational interventions, exercise, weight reduction, various classes of analgesics, nonsteroidal anti-inflammatory drugs, muscle relaxants, antidepressants, behavioral therapy, physical therapy, spinal manipulation, other complementary and alternative therapies, and surgery [11,12].

The purpose of this study was to elucidate the epidemiology and medical management of LBP during ambulatory medical care visits in the United States.

## Methods

### Overview of the National Ambulatory Medical Care Survey design

The concept of the National Ambulatory Medical Care Survey (NAMCS) to collect data on medical care provided in physician offices in the United States was developed over 30 years ago [13]. Detailed documentation of the NAMCS instrument, methodology, and data files that served as the basis for this study is available elsewhere [14,15]. Patient visits were selected using a multistage probability sample design. The first stage included primary sampling units (PSUs) which consisted of counties, groups of counties, county equivalents (e.g. parishes), towns, townships, minor civil divisions, or metropolitan statistical areas (MSAs). These PSUs comprised a probability subsample of those used in the 1985–1994 National Health Interview Surveys [16]. The latter, which covered all 50 states and the District of Columbia, were stratified by demographic and socioeconomic variables and then selected with probability proportional to their size. Stratification was done within four geographic regions by MSA and non-MSA status.

The second stage consisted of a probability sample of practicing physicians selected from the master files of the American Medical Association (AMA) and American Osteopathic Association (AOA). Within each PSU, all eligible physicians were further stratified by specialty. The third stage involved selection of patient visits within the practices of participating physicians. Initially, physicians were randomly assigned to one of the 52 weeks within a calendar year. Then, a systematic random sample of patient visits was selected for each physician during the assigned week. The sampling rate of patient visits varied from a 20% sample for very large practices to 100% for very small practices as determined by a presurvey interview [17]. In this manner, data from about 30 patient visits were recorded by each physician during the assigned week.

### Sampling frame and sample size

The sampling frame for NAMCS included physicians who met the criteria of being: (1) office-based; (2) principally engaged in patient care activities; (3) nonfederally employed; and (4) not in the specialties of anesthesiology, pathology, or radiology. During 2003 and 2004, a total of 6000 physicians were initially screened. Of these, 2032 (34%) did not meet the four inclusion criteria, most commonly because the physician was retired, deceased, or employed in teaching, research, or administration. Of the remaining 3968 eligible physicians, 2779 (70%) participated in NAMCS. However, among these "participating" physicians, 544 (20%) saw no patients during their assigned reporting period because of vacations, illness, or other reasons for being temporarily not in practice. The NAMCS provides data on 25,288 patient visits to 1114 physician offices during the 2003 calendar year and 25,286 patient visits to 1121 physician offices during the 2004 calendar year.

### Patient visits and weights

The basic sampling unit for the NAMCS is the physician-patient encounter or "patient visit." The following types of contacts were excluded: telephone calls, visits outside the physician's office (e.g., house calls), visits made in hospital settings (unless the physician had a private office in a hospital), visits made in institutional settings that had primary responsibility for the patient's care (e.g., nursing homes), and visits to the physician's office for administrative purposes only (e.g., to leave a specimen, pay a bill, or pick up insurance forms). Each patient visit was assigned a weight based on four factors: (1) probability of being selected by the three-stage sampling design; (2) adjustment for nonresponse; (3) adjustment for physician specialty group; and (4) weight smoothing to minimize the impact of a few physician outliers whose final visit weights were large relative to those for the remaining physicians. Thus, by applying these weights to each of the 50,574 patient visits in the 2003 and 2004 NAMCS data files, an estimated 1.8 billion physician office visits in the United States were available to derive unbiased national estimates of ambulatory medical care services and to further characterize such services.

### Data collection and processing

Data for the NAMCS were collected by the physician with assistance from office staff when possible. Patient record forms were used to collect the data for each selected visit. The NAMCS field staff performed completeness checks of the patient record forms prior to submission for central data processing. Detailed editing instructions were provided to reclassify or recode ambiguous or inconsistent data entries. Quality control measures, which were used to verify the accuracy of computer data entry, indicated that the mean keying error rate was 0.1% for nonmedical items and that discrepancy rates ranged from 0.0% to 1.1% for required medical items.

Item nonresponse rates were 5% or less for most variables. Major exceptions (nonresponse rate) included: ethnicity (20%), race (18%), tobacco use (30%), and time spent with physician (16%). Missing data for birth year (4%), sex (4%), race (18%), ethnicity (20%), and time spent with physician (16%) were imputed by assigning the value from a randomly selected patient record form representing another patient with similar known characteristics. Such imputations were performed according to physician specialty, geographic region (state was used instead of geographic region to impute ethnicity), and primary diagnosis codes.

### Data management and statistical analyses

This study focused on patient visits for LBP. These were initially identified using the "reason for visit" (RFV) item of the NAMCS patient record form. Specifically, patient visits were included only if back symptoms (RFV classification code number, 1905) or low back symptoms (RFV classification code number, 1910) were reported as one of the three most important reasons for the visit in the patient's own words. Subsequently, any patient visits attributed to a lump, mass, or tumor of the back or low back were excluded. Exploratory analyses of the data stratified according to RFV classification code numbers (1905 vs. 1910) and importance of symptoms (primary reason for visit vs. secondary or tertiary reason for visit) revealed few substantive differences between groups. Consequently, to maximize statistical power, all epidemiological analyses combined RFV classification code numbers 1905 and 1910 to represent prevalent cases of LBP (i.e., back symptoms, other than those attributed to a lump, mass, or tumor, were the primary, secondary, or tertiary reason for the patient visit). However, only those patient visits in which LBP was the primary reason for seeking medical care ("primary LBP patient visits") were included in the medical management analyses to minimize potential confounding by other secondary or tertiary reasons for the patient visit. Patient visits attributed to neck symptoms (RFV classification code number, 1900) exclusive of LBP were not included in the study. National population estimates derived from the NAMCS may be unreliable if they are based on fewer than 30 unweighted patient visits or if the relative standard error (standard error divided by the national population estimate) is greater than 0.30 [14,15].

Patient sociodemographic characteristics included age, sex, race, ethnicity, geographic region, and MSA status of residence. Patient visit context characteristics included episode of care, chronicity of symptoms, and whether the visit was related to an injury, poisoning, or adverse effect (IPA). Physician provider characteristics included primary care physician status, type of physician provider (Doctor of Medicine or Doctor of Osteopathy), physician specialty, and whether multiple physicians shared responsibility for medical care of the patient. The elements of LBP medical management included any diagnostic tests, patient counseling, drugs, physiotherapy, or surgical procedures that were ordered, scheduled, or performed during the patient visit. Drugs were broadly defined as any medications or injections, including immunizations, allergy shots, anesthetics, or dietary supplements, that were ordered, supplied, administered, or continued during the visit, regardless of prescription or over-the-counter status. Up to eight drugs may have been listed on the NAMCS patient record form during a patient visit. For each drug listed, up to three therapeutic class codes were assigned based on the standard classifications used in the National Drug Code (NDC) Directory [18]. The drugs portion of this study focused exclusively on three common drugs for relief of pain (NDC code, 1700): (1) non-narcotic analgesics (NDC code, 1722); (2) narcotic analgesics (NDC code, 1721); and (3) nonsteroidal anti-inflammatory drugs (NSAIDs) (NDC code, 1727), including antiarthritics (NDC code, 1724). To maintain consistency with the NDC codes used in the NAMCS patient record form, the term "narcotic analgesic" will be used herein rather than "opioid." Physiotherapy consisted of treatments using heat, light, sound, physical pressure, or movement, including manipulative therapy.

To study the epidemiology of LBP, national population estimates of patient visits were derived and stratified according to patient sociodemographic, patient visit context, and physician provider characteristics. Multiple logistic regression was used to compute adjusted odds ratios (ORs) and 95% confidence intervals (CIs) for factors associated with LBP patient visits compared with patient visits for all other reasons. Similar analyses were repeated according to chronicity of LBP: (1) initial visits for acute LBP and (2) follow-up visits for chronic LBP. To study the LBP medical management, national population estimates of the use of diagnostic tests, patient counseling, drugs, physiotherapy, and surgical procedures were derived for patient visits in which LBP was the primary reason for seeking medical care. Simple logistic regression was initially performed to compute crude ORs and 95% CIs for the elements of chronic LBP medical management compared with acute LBP medical management. Multiple logistic regression was subsequently used to compute adjusted ORs and 95% CIs for the most commonly used elements of LBP medical management. All hypotheses were tested at the .05 level of statistical significance.

The electronic files containing the 2003 and 2004 NAMCS data were acquired from the National Center for Health Statistics. The files were merged and analyzed using SPSS Version 14.0 for Windows (SPSS Inc., Chicago, IL). Because the multistage probability design of the NAMCS includes clustering, stratification, and the assignment of unequal probabilities of selection to sample units, all analyses were performed with the SPSS complex samples module to accurately compute estimates of population parameters and their standard errors [19]. A check of these statistical procedures, which involved the entire 2003 and 2004 NAMCS databases, verified that the computed marginal totals for national population estimates were identical to those published by the National Center for Health Statistics [14,15].

## Results

### National population estimates of patient visits for low back pain

**Figure 1:**
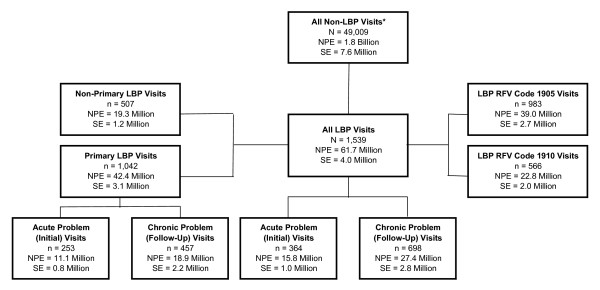
National population estimates of patient visits for low back pain in the United States, 2003–2004. LBP indicates low back pain; NPE, national population estimate; SE, standard error. National population estimates are for both years combined and were computed by applying patient visits weights to the numbers reported in the National Ambulatory Medical Care Survey. Patient visits involving back symptoms attributed to a lump, mass, or tumor are not included as LBP visits. Patient visits other than initial visits for acute problems or follow-up visits for chronic problems are not represented under the "all LBP visits" and "primary LBP visits" headings.

There were an estimated 31 million (3%) patient visits annually attributed to LBP in the United States. For the 2003–2004 period, 1539 patient record forms representing 61.7 million (SE, 4.0 million) LBP patient visits and 1042 patient record forms representing 42.4 million (SE, 3.1 million) primary LBP patient visits were included in the analyses reported herein (Figure 1). The physician specialties most commonly seen during LBP patient visits were family (general) medicine, 25.2 million (SE, 2.3 million); internal medicine, 14.4 million (SE, 3.0 million); and orthopedics, 5.6 million (SE, 0.9 million). In orthopedics, one of every 16 patient visits involved LBP (one of every 11 working-age patients seen for reasons other than preventive or surgery-related care). In family (general) medicine, one of every 17 patient visits involved LBP (one of every 13 working-age patients seen for reasons other than preventive or surgery-related care). Patient visits for LBP were attributed to the following factors: (1) back pain, ache, soreness, or discomfort, 94%; (2) other back symptoms, including cramps, contractures, spasms, limitation of movement or stiffness, or weakness, 2%; and (3) other unspecified back symptoms, 4%. Prior to combining LBP patient visits, they were originally designated as RFV classification code number 1905 in 39.0 million (SE, 2.7 million) patient visits and as RFV classification code number 1910 in 22.8 million (SE, 2.0 million) patient visits. A total of 42.4 million (SE, 3.1 million) patient visits (69%) involved LBP as the primary reason for seeking medical care.

There were few differences in patient visit characteristics according to the importance of LBP (primary reason for seeking medical care vs. secondary or tertiary reasons for seeking medical care) or RFV classification code number (1905 vs. 1910) (Table 1). Neurologists were less likely than other physician specialties to provide care for primary LBP (P = .05). Hispanics were more likely than non-Hispanics (P = .02) and osteopathic physicians were more likely than allopathic physicians (P = .04) to involve RFV classification code number 1910 rather than 1905.

**Table 1:** Patient Visits for Low Back Pain According to Importance and Classification of Reason for Visit, 2003–2004 (N = 1,539)*

	**Importance of Reason for Visit**								**Reason for Visit Classification Code†**							
		
	**Primary**				**Secondary or Tertiary**				**1905**				**1910**			
				
**Characteristic**	**n**	**NPE**	**SE**	**%**	**n**	**NPE**	**SE**	**%**	**n**	**NPE**	**SE**	**%**	**n**	**NPE**	**SE**	**%**
**Patient age, y**																
<25	73	2,828	420	7	42	1,595	316	8	81	3,257	430	8	34	1,167	274	5
25–44	340	14,052	1,194	33	133	5,308	553	28	289	11,598	1,091	30	184	7,763	666	34
45–64	392	16,122	1,796	38	206	7,476	934	39	364	14,192	1,279	36	234	9,406	1,432	41
≥ 65	237	9,424	1,118	22	126	4,910	620	25	249	9,918	1,209	25	114	4,417	655	19
																
**Patient sex**																
Female	589	24,275	1,941	57	319	12,377	875	64	582	23,178	1,852	59	326	13,474	1,074	59
Male	453	18,152	1,716	43	188	6,913	749	36	401	15,786	1,312	41	240	9,279	1,277	41
																
**Patient race**																
White	936	37,791	2,951	89	439	16,567	1,140	86	870	33,905	2,480	87	505	20,453	1,815	90
Non-White	106	4,635	637	11	68	2,724	604	14	113	5,059	924	13	61	2,300	366	10
																
**Patient ethnicity**																
Non-Hispanic	943	38,746	3,055	91	458	17,455	1,155	90	903	36,011	2,604	92	498	20,189	1,821	89
Hispanic	99	3,681	509	9	49	1,836	378	10	80	2,953	484	8	68	2,563	395	11
																
**Geographic region**																
Northeast	292	13,189	2,211	31	149	5,376	760	28	253	10,157	1,582	26	188	8,408	1,689	37
Midwest	222	8,102	1,146	19	107	3,602	400	19	216	7,966	1,170	20	113	3,739	447	16
South	313	11,984	1,319	28	146	5,964	607	31	315	12,592	1,411	32	144	5,356	558	24
West	215	9,152	1,313	22	105	4,348	580	23	199	8,249	1,090	21	121	5,250	683	23
																
**MSA status**																
MSA	861	36,579	3,208	86	432	17,125	1,344	89	815	33,342	2,882	86	478	20,362	2,034	89
Non-MSA	181	5,848	1,653	14	75	2,166	599	11	168	5,623	1,497	14	88	2,391	652	11
																
**Episode of care**																
Initial	351	14,286	1,126	34	158	6,210	614	32	322	13,279	1,093	34	187	7,217	743	32
Follow-up	617	24,754	2,483	58	304	10,893	1,011	56	586	22,309	1,962	57	335	13,338	1,699	59
Other or missing	74	3,387	545	8	45	2,188	373	11	75	3,376	543	9	44	2,198	371	10
																
**Chronicity of symptoms**																
Acute	402	16,819	1,260	40	169	6,632	685	34	360	14,820	1,292	38	211	8,631	712	38
Chronic, routine	398	16,797	2,342	40	211	7,096	845	37	380	14,874	1,685	38	229	9,019	1,603	40
Chronic, flare-up	176	6,494	779	15	79	2,990	404	15	171	6,280	724	16	84	3,203	513	14
Other or missing‡	66	2,317	402	5	48	2,573	471	13	72	2,990	388	8	42	1,900	340	8
																
**IPA etiology**																
No	722	28,796	2,161	68	341	13,215	1,006	69	698	27,443	2,195	70	365	14,568	1,201	64
Yes	320	13,631	1,449	32	166	6,075	716	31	285	11,522	1,040	30	201	8,185	1,100	36
																
**PCP status**																
Non-PCP or unknown	589	18,744	2,306	44	314	9,241	918	48	532	16,465	1,940	42	371	11,519	1,564	51
PCP	453	23,683	1,845	56	193	10,049	958	52	451	22,499	1,823	58	195	11,233	934	49
																
**Type of physician**																
Doctor of Medicine	782	31,845	2,192	75	407	15,399	1,080	80	762	30,832	2,257	79	427	16,412	1,132	72
Doctor of Osteopathy	260	10,582	2,130	25	100	3,892	600	20	221	8,132	1,434	21	139	6,341	1,631	28
																
**Physician specialty**																
Family (general) medicine	404	18,523	1,737	44	145	6,687	692	35	382	17,484	1,879	45	167	7,725	1,011	34
Internal medicine	107	9,702	2,264	23	54	4,703	1,008	24	96	8,199	1,257	21	65	6,206§	2,234	27
Neurology	141	1,244	218	3	96	1,011	213	5	132	1,138	181	3	105	1,116	322	5
Orthopedics	151	3,618	546	9	66	2,020	489	10	121	3,025	478	8	96	2,613	505	11
All other specialties	239	9,340	1,278	22	146	4,869	586	25	252	9,118	1,160	23	133	5,092	697	22
																
**Shared physician care**																
No or unknown	715	31,694	2,633	75	336	13,417	1,007	70	690	28,951	2,184	74	361	16,160	1,692	71
Yes	327	10,733	1,017	25	171	5,873	672	30	293	10,013	963	26	205	6,593	703	29
																
**Total**	**1,042**	**42,427**	**3,108**	**100**	**507**	**19,290**	**1,201**	**100**	**983**	**38,964**	**2,655**	**100**	**566**	**22,753**	**1,957**	**100**

Abbreviations: IPA, injury, poisoning, or adverse effect of medical treatment; MSA, metropolitan statistical area; NPE, national population estimate for the two-year interval; PCP, primary care physician, SE, standard error of the national population estimate.

*Table entries for NPE and SE are in thousands; percentages are based on NPE.

†1905 represents back pain and 1910 represents low back pain.

‡Other options included pre- or post-surgery visits, prenatal care, and health maintenance visits.

§NPE may be unreliable because SE>30% of NPE.

### The epidemiology of ambulatory medical care visits for low back pain

Patient visits for LBP predominantly involved persons aged 25 to 64 years (69%) and females (59%) (Table 2). A majority of patient visits were for follow-up care. Patient visits were about evenly divided between acute and routine chronic LBP, and about one-third of visits were attributed to an IPA. Among the estimated 8.4 million patient visits in which an IPA was specified, 7.4 million (88%) involved injuries (primarily overuse syndromes, motor vehicle accidents, or falls) and 0.9 million (11%) involved adverse effects of surgical procedures, drugs, or environmental agents. Thus, IPAs served as a useful surrogate for injuries. Only slightly more than half (55%) of the patient visits were provided by primary care physicians.

**Table 2:** Patient Visits According to Presence or Absence of Low Back Pain, 2003–2004 (N = 50,558)*

	**LBP Status**											
					
	**LBP Present**				**LBP Absent**				**LBP Visits**		**Primary LBP Visits†**	
				
**Characteristic**	**n**	**NPE**	**SE**	**%**	**n**	**NPE**	**SE**	**%**	**OR‡**	**95% CI**	**OR‡**	**95% CI**
**Patient age, y**												
<25	115	4,423	572	7	10,764	431,753	24,165	25	0.30	0.22 0.41	0.26	0.18 – 0.38
25–44	473	19,360	1,410	31	10,212	378,110	19,956	22	1.00	...	1.00	...
45–64	598	23,599	2,409	38	14,426	497,737	25,996	28	0.84	0.71 – 1.00	0.78	0.65 – 0.95
≥ 65	363	14,335	1,443	23	13,607	447,079	23,110	25	0.60	0.47 – 0.75	0.54	0.41 – 0.70
												
**Patient sex**												
Female	908	36,652	2,539	59	27,581	1,035,915	46,027	59	1.00	...	1.00	...
Male	641	25,065	2,042	41	21,428	718,765	32,578	41	0.93	0.81 – 1.06	1.01	0.85 – 1.20
												
**Patient race**												
White	1,375	54,358	3,825	88	42,716	1,497,330	71,034	85	1.00	...	1.00	...
Non-White	174	7,359	1,015	12	6,293	257,349	16,925	15	0.88	0.68 – 1.14	0.80	0.61 – 1.06
												
**Patient ethnicity**												
Non-Hispanic	1,401	56,201	3,846	91	44,432	1,565,163	68,318	89	1.00	...	1.00	...
Hispanic	148	5,516	676	9	4,577	189,516	22,488	11	1.02	0.81 – 1.29	1.01	0.76 – 1.34
												
**Geographic region**												
Northeast	441	18,566	2,856	30	10,111	340,535	31,296	19	1.00	...	1.00	...
Midwest	329	11,704	1,608	19	11,056	367,864	38,458	21	0.56	0.40 – 0.77	0.53	0.37 – 0.75
South	459	17,948	1,584	29	17,262	674,752	49,514	38	0.56	0.40 – 0.77	0.52	0.36 – 0.75
West	320	13,499	1,630	22	10,580	371,528	29,742	21	0.75	0.52 – 1.07	0.71	0.48 – 1.06
												
**MSA status**												
MSA	1,293	53,704	4,318	87	42,768	1,534,434	99,888	87	1.00	...	1.00	...
Non-MSA	256	8,013	2,093	13	6,241	220,245	51,084	13	1.00	0.78 – 1.28	1.06	0.79 – 1.43
												
**Episode of care**												
Initial	509	20,495	1,431	33	14,004	526,779	23,102	30	1.00	...	1.00	...
Follow-up	921	35,647	3,256	58	26,324	829,669	41,893	47	0.98	0.83 – 1.16	1.02	0.83 – 1.26
Other or missing	119	5,575	706	9	8,681	398,231	23,040	23	0.80	0.56 – 1.14	0.88	0.57 – 1.36
												
**Chronicity of symptoms**												
Acute	571	23,451	1,602	38	15,741	615,627	25,898	35	1.00	...	1.00	...
Chronic, routine	609	23,893	2,932	39	17,885	561,484	30,854	32	1.37	1.08 – 1.74	1.36	1.03 – 1.79
Chronic, flare-up	255	9,483	985	15	4,493	139,324	9,200	8	2.09	1.65 – 2.65	2.03	1.58 – 2.63
Other or missing§	114	4,890	618	8	10,890	438,244	24,905	25	0.52	0.35 – 0.77	0.33	0.20 – 0.56
												
**IPA etiology**												
No	1,063	42,011	2,824	68	43,823	1,569,266	68,357	89	1.00	...	1.00	...
Yes	486	19,707	1,785	32	5,186	185,413	10,269	11	3.38	2.75 – 4.14	3.33	2.64 – 4.21
												
**PCP status**												
Non-PCP or unknown	903	27,985	2,959	45	33,809	925,652	54,734	53	1.00	...	1.00	...
PCP	646	33,732	2,345	55	15,200	829,027	38,795	47	0.84	0.61 – 1.16	0.82	0.55 – 1.21
												
**Type of physician**												
Doctor of Medicine	1,189	47,244	2,859	77	45,316	1,636,480	72,120	93	1.00	...	1.00	...
Doctor of Osteopathy	360	14,474	2,718	23	3,693	118,199	12,647	7	2.61	1.75 – 3.92	2.68	1.77 – 4.06
												
**Physician specialty**												
Family (general) medicine	549	25,210	2,259	41	8,240	405,563	26,625	23	3.28	2.26 – 4.77	3.59	2.26 – 5.72
Internal medicine	161	14,405	3,074	23	3,044	273,218	23,834	16	3.34	2.11 – 5.27	3.38	1.95 – 5.85
Neurology	237	2,255	405	4	3,202	25,403	2,685	1	3.97	2.69 – 5.85	3.25	2.07 – 5.08
Orthopedics	217	5,638	896	9	2,521	82,010	9,469	5	2.27	1.48 – 3.49	2.19	1.35 – 3.53
All other specialties	385	14,209	1,688	23	32,002	968,485	49,844	55	1.00	...	1.00	...
												
**Shared physician care**												
No or unknown	1,051	45,111	3,282	73	35,679	1,339,868	60,278	76	1.00	...	1.00	...
Yes	498	16,606	1,413	27	13,330	414,812	22,483	24	1.17	0.99 – 1.39	1.09	0.88 – 1.35
												
**Total**	**1,549**	**61,717**	**3,988**	**100**	**49,009**	**1,754,679**	**76,123**	**100**		...		...

Abbreviations: CI, confidence interval; IPA, injury, poisoning, or adverse effect of medical treatment; LBP, low back pain; MSA, metropolitan statistical area; NPE, national population estimate for the two-year interval; OR, odds ratio; PCP, primary care physician, SE, standard error of the national population estimate.

*Table entries for NPE and SE are in thousands; percentages are based on NPE.

†Includes only patient visits in which LBP was the primary reason for seeking medical care (n = 1042).

‡Adjusted for all variables shown.

§Other options included pre- or post-surgery visits, prenatal care, and health maintenance visits.

When compared with the 1.8 billion (SE, 7.6 million) patient visits for reasons other than back symptoms, LBP visits were associated with several factors (Table 2). Low back pain was less likely the reason for a patient visit in all younger and older age categories compared with the referent category (25–44 years) (P < .001). There was a geographic variation in patient visits attributed to LBP (P = .001), with fewer visits in the Midwest and South than in the Northeast. Patient visits for LBP were more likely to reflect chronicity of symptoms, either routine ongoing problems or flare-ups, than were patient visits for other reasons (P < .001). However, injuries, as reflected by the surrogate IPA item, were important predictors of LBP patient visits (P < .001). The type of physician provider (P < .001) and physician specialty (P < .001) were associated with LBP patient visits, with osteopathic physicians, family (general) medicine physicians, internal medicine physicians, neurologists, and orthopedic surgeons being more likely to provide medical care during such visits. Similar findings were observed when the analyses involved only primary LBP patient visits rather than all LBP patient visits.

A total of 15.8 million (SE, 1.0 million) LBP patient visits were initial visits for an acute problem (less than three months since onset) and 27.4 million (SE, 2.8 million) LBP patient visits were follow-up visits for chronic LBP (Table 3). Age <25 years (P < .001), injury (P < .001), and being seen by a primary care physician (P = .01) were inversely associated with LBP chronicity, whereas being seen by an osteopathic physician (P < .001) and shared physician care (P = .003) were directly associated with LBP chronicity. Again, similar findings were observed when the analyses involved only primary LBP patient visits rather than all LBP patient visits.

**Table 3:** Patient Visits for Low Back Pain According to Chronicity, 2003–2004 (n = 1,062)*

	**Chronicity**											
					
	**Acute Problem** **(Initial Visit)**				**Chronic Problem** **(Follow-Up Visit)**				**Chronic Problem Visits**		**Primary Chronic Problem Visits†**	
				
**Characteristic**	**n**	**NPE**	**SE**	**%**	**n**	**NPE**	**SE**	**%**	**OR‡**	**95% CI**	**OR‡**	**95% CI**
**Patient age, y**												
<25	49	2,012	287	13	26	836§	197	3	0.27	0.13-0.55	0.20	0.09-0.45
25–44	112	5,351	565	34	195	7,920	907	29	1.00	...	1.00	...
45–64	123	5,157	597	33	287	11,025	1,867	40	1.27	0.84-1.93	1.24	0.81-1.91
≥ 65	80	3,237	542	21	190	7,585	1,110	28	1.42	0.84-2.40	1.29	0.66-2.50
												
**Patient sex**												
Female	218	9,242	757	59	400	15,815	1,684	58	1.00	...	1.00	...
Male	146	6,515	775	41	298	11,551	1,463	42	1.17	0.79-1.73	1.22	0.81-1.85
												
**Patient race**												
White	329	13,955	966	89	619	24,498	2,821	90	1.00	...	1.00	...
Non-White	35	1,802	404	11	79	2,867	554	10	1.03	0.60-1.76	0.85	0.45-1.60
												
**Patient ethnicity**												
Non-Hispanic	326	14,164	984	90	642	25,299	2,718	92	1.00	...	1.00	...
Hispanic	38	1,593	334	10	56	2,066	386	8	0.74	0.42-1.32	0.86	0.41-1.77
												
**Geographic region**												
Northeast	91	3,494	363	22	211	9,671	2,386	35	1.00	...	1.00	...
Midwest	76	3,189	495	20	133	3,918	452	14	0.65	0.40-1.08	0.66	0.33-1.32
South	131	5,775	670	37	201	7,569	848	28	0.86	0.52-1.40	0.90	0.51-1.61
West	66	3,299	460	21	153	6,207	1,214	23	1.23	0.62-2.46	1.03	0.45-2.38
												
**MSA status**												
MSA	307	13,706	1,113	87	588	24,113	2,917	88	1.00	...	1.00	...
Non-MSA	57	2,050	572	13	110	3,252	973	12	1.16	0.67-1.99	1.05	0.58-1.92
												
**IPA etiology**												
No	224	9,611	860	61	518	20,345	1,874	74	1.00	...	1.00	...
Yes	140	6,146	586	39	180	7,020	1,350	26	0.46	0.31-0.67	0.49	0.32-0.75
												
**PCP status**												
Non-PCP or unknown	170	4,733	518	30	437	13,841	2,151	51	1.00	...	1.00	...
PCP	194	11,024	963	70	261	13,524	1,366	49	0.45	0.26-0.79	0.48	0.25-0.90
												
**Type of physician**												
Doctor of Medicine	299	13,656	932	87	497	18,256	1,623	67	1.00	...	1.00	
Doctor of Osteopathy	65	2,101	285	13	201	9,109	2,402	33	4.39	2.47-7.80	4.08	2.26-7.36
												
**Physician specialty**												
Family (general) medicine	163	7,990	768	51	227	10,248	1,341	37	0.58	0.31-1.07	0.64	0.31-1.32
Internal medicine	42	3,611	560	23	80	7162§	2,603	26	0.97	0.48-1.95	0.98	0.40-2.39
Neurology	36	287	63	2	129	1,283	262	5	1.41	0.67-2.94	0.91	0.34-2.45
Orthopedics	44	1,064	166	7	81	2,279	407	8	0.89	0.48-1.65	1.17	0.50-2.73
All other specialties	79	2,804	372	18	181	6,394	1,096	23	1.00	...	1.00	...
												
**Shared physician care**												
No or unknown	283	13,096	1,008	83	448	19,240	2,565	70	1.00	...	1.00	...
Yes	81	2,661	401	17	250	8,125	960	30	2.11	1.30-3.44	2.15	1.18-3.93
												
**Total**	**364**	**15,757**	**1,018**	**100**	**698**	**27,365**	**2,844**	**100**		...		...

Abbreviations: CI, confidence interval; IPA, injury, poisoning, or adverse effect of medical treatment; MSA, metropolitan statistical area; NPE, national population estimate for the two-year interval; OR, odds ratio; PCP, primary care physician, SE, standard error of the national population estimate.

*Table entries for NPE and SE are in thousands; percentages are based on NPE.

†Includes only patient visits in which low back pain was the primary reason for seeking medical care (n = 710).

‡Adjusted for all variables shown.

§NPE may be unreliable because n<30 or SE>30% of NPE.

### The medical management of primary low back pain during ambulatory medical care visits

**Figure 2:**
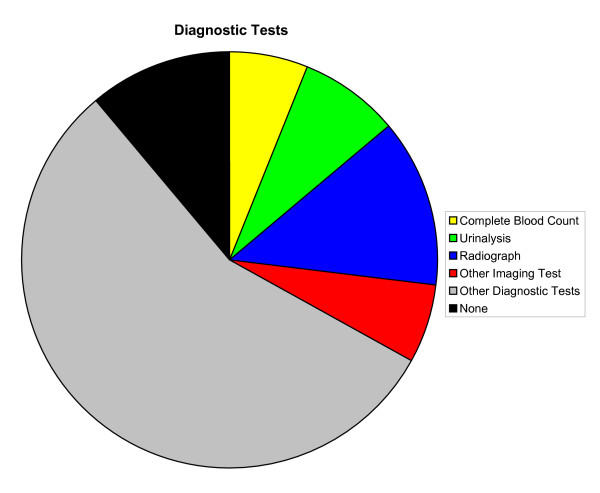
Diagnostic tests in the medical management of primary low back pain in the United States, 2003–2004. Other imaging tests are exclusive of mammograms.

**Figure 3:**
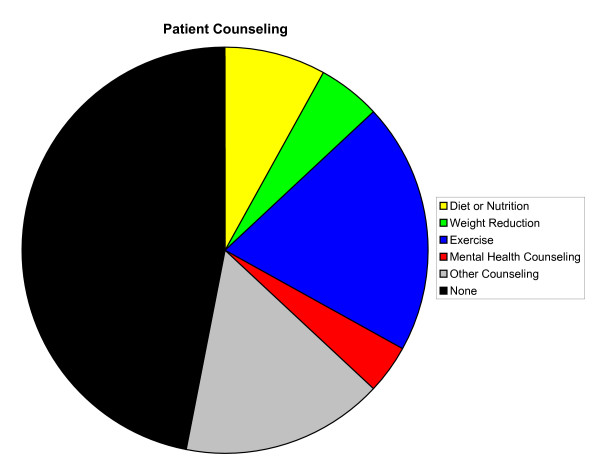
Patient counseling in the medical management of primary low back pain in the United States, 2003–2004. Mental health counseling also includes stress management and psychotherapy.

**Figure 4:**
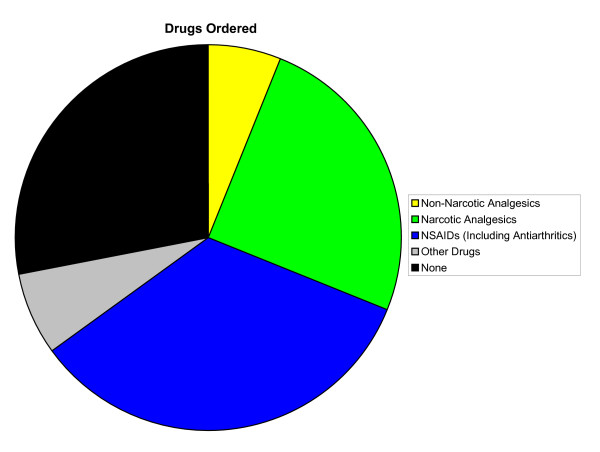
Drugs ordered in the medical management of primary low back pain in the United States, 2003–2004. NSAID indicates nonsteroidal anti-inflammatory drug.

Overall, a majority of primary LBP patient visits included diagnostic tests (89%), patient counseling (53%), and orders for drugs (72%) (Figures 2, 3, 4, respectively). Compared with patient visits in which LBP was absent, primary LBP patient visits were more likely to involve patient counseling (OR, 1.54; 95% CI, 1.17–2.04) and physiotherapy (OR, 7.89; 95% CI, 6.01–10.35); however, they were less likely to involve surgical procedures (OR, 0.56; 95% CI, 0.38–0.83). There were no significant differences in the frequency of diagnostic tests performed or drugs ordered. The most common elements of LBP medical management included radiographs (13%), exercise counseling (20%), NSAIDs (34%), narcotic analgesics (25%), and physiotherapy (20%). There were 14.2 million (SE, 1.2 million) and 10.5 million (SE, 1.1 million) orders, respectively, for NSAIDs and narcotic analgesics during these primary LBP patient visits (Table 4). Except for the variables "exercise counseling," "any patient counseling," "narcotic analgesics," "NSAIDs," and "any drug ordered," the elements of primary LBP medical management were not reported in sufficiently high numbers of patient visits to provide statistically valid and reliable comparisons of initial visits for acute LBP and follow-up visits for chronic LBP. Drugs generally (P = .002), and NSAIDs specifically (P < .001), were ordered less often during follow-up visits for chronic LBP than during initial visits for acute LBP. Similarly, diagnostic tests were generally performed less often during follow-up visits for chronic LBP than during initial visits for acute LBP (P = .003). However, surgical procedures were ordered more often during follow-up visits for chronic LBP than during initial visits for acute LBP (P < .001).

**Table 4:** Medical Management during Patient Visits in which Low Back Pain was the Primary Reason for Seeking Care, 2003–2004 (n = 1,042)*

					**Chronicity**									
							
	**LBP Visits**				**Acute Problem** **(Initial Visit)**				**Chronic Problem** **(Follow-Up Visit)**				**Chronic Problem Visits**	
				
**Medical Management**	**n**	**NPE**	**SE**	**%**	**n**	**NPE**	**SE**	**%**	**n**	**NPE**	**SE**	**%**	**OR**	**95% CI**
**Diagnostic tests**														
Complete blood count	52	2,663	416	6	12	525	197	5	19	1,165	265	6	1.32	0.52-3.37
Urinalysis	69	3,243	456	8	39	1,754	359	16	10	613	161	3	0.18	0.09-0.37
Radiograph	142	5,602	634	13	57	2,385	406	21	24	830	195	4	0.17	0.09-0.32
Other imaging test†	83	2,434	345	6	24	632	146	6	29	904	191	5	0.83	0.44-1.58
***Any diagnostic test***	926	37,683	2,608	89	244	10,807	809	97	396	15,734	1,419	83	0.14	0.04-0.49
														
**Patient counseling**														
Diet or nutrition	75	3,335	602	8	15	734	196	7	40	1,688	488	9	1.38	0.58-3.32
Weight reduction	49	2,232	529	5	10	504	183	5	25	1,143	384	6	1.35	0.52-3.49
Exercise	204	8,320	1,070	20	49	2,196	391	20	95	3,979	771	21	1.08	0.63-1.85
Mental health counseling‡	45	1,859	639	4	5	240	124	2	31	1,176	525	6	3.00	0.94-9.61
***Any patient counseling***	513	22,397	2,510	53	119	5,408	563	49	224	10,138	1,956	54	1.21	0.70-2.11
														
**Drugs**														
Non-narcotic analgesics	58	2,492	431	6	13	669	201	6	29	1,351	301	7	1.20	0.51-2.82
Narcotic analgesics	261	10,503	1,128	25	56	2,385	444	21	141	5,355	758	28	1.44	0.76-2.72
NSAIDs (including antiarthritics)	333	14,237	1,180	34	113	5,497	596	49	128	5,288	667	28	0.40	0.25-0.64
***Any drug***	729	30,523	2,012	72	204	9,746	798	88	316	12,703	1,233	67	0.29	0.13-0.62
														
**Physiotherapy§**	179	8,596	2,135	20	32	1,408	325	13	88	4,866	1,887	26	2.38	0.93-6.07
														
**Surgical procedure**	59	1,932	407	5	2	51	35	0	27	1,042	304	6	12.62	3.18-50.07
														
**Total**	**1,042**	**42,427**	**3,108**	**100**	**253**	**11,111**	**798**	**100**	**457**	**18,948**	**2,154**	**100**		...

Abbreviations: CI, confidence interval; LBP, low back pain; NPE, national population estimate for the two-year interval; NSAID, nonsteroidal anti-inflammatory drug; OR, odds ratio; SE, standard error of the national population estimate.

*Table entries for NPE and SE are in thousands; percentages are based on NPE. NPEs may not be reliable for variables other than any diagnostic test, any patient counseling, any drug, exercise counseling, NSAIDs, and narcotic analgesics because n<30 and/or SE>30% of NPE.

†Excluding mammograms.

‡Including mental health counseling, stress management, or psychotherapy.

§Including spinal manipulation.

Several other factors emerged in association with the common elements of primary LBP medical management after controlling for potential confounders (Table 5). Patient counseling was less often provided for non-Whites (P = .04) and in geographic regions outside the Northeast (P = .01), although it was provided more often when injuries were reported (P < .001). Specifically, with regard to exercise counseling, there remained a geographic variation (P = .003) and propensity for use following injury (P < .001). However, exercise counseling was less often provided in non-MSAs (P = .04) and by various physician specialties (P < .001), including family (general) medicine physicians and internal medicine physicians.

**Table 5:** Factors Associated with Medical Management during Patient Visits in which Low Back Pain was the Primary Reason for Seeking Care, 2003–2004 (n = 710)*

	**Medical Management**									
	
	**Exercise Counseling**		**Any Patient Counseling**		**Narcotic Analgesic**		**NSAID**		**Any Drug**	
					
**Characteristic**	**OR†**	**95% CI**	**OR†**	**95% CI**	**OR†**	**95% CI**	**OR†**	**95% CI**	**OR†**	**95% CI**
**Patient age, y**										
<25	0.53	0.15 – 1.90	0.64	0.27 – 1.53	0.35	0.11 – 1.11	0.93	0.44·1.94	0.19	0.08 – 0.50
25–44	1.00	...	1.00	...	1.00	...	1.00	...	1.00	...
45–64	1.20	0.66 – 2.19	1.08	0.66 – 1.75	0.80	0.47 – 1.36	0.64	0.39·1.06	0.63	0.36 – 1.09
≥65	0.93	0.47 – 1.83	0.67	0.38 – 1.19	0.42	0.21 – 0.84	0.73	0.44·1.20	0.77	0.38 – 1.56
										
**Patient sex**										
Female	1.00	...	1.00	...	1.00	...	1.00	...	1.00	...
Male	0.85	0.52 – 1.38	0.91	0.62 – 1.36	0.90	0.59 – 1.39	0.99	0.68·1.45	0.76	0.50 – 1.14
										
**Patient race**										
White	1.00	...	1.00	...	1.00	...	1.00	...	1.00	...
Non-White	0.66	0.23 – 1.89	0.49	0.25 – 0.96	0.91	0.42 – 2.01	1.30	0.60·2.84	3.54	1.40 – 8.97
										
**Patient ethnicity**										
Non-Hispanic	1.00	...	1.00	...	1.00	...	1.00	...	1.00	...
Hispanic	1.45	0.77 – 2.77	1.45	0.77 – 2.77	0.30	0.11 – 0.76	1.12	0.53·2.36	1.78	0.73 – 4.36
										
**Geographic region**										
Northeast	1.00	...	1.00	...	1.00	...	1.00	...	1.00	...
Midwest	0.33	0.14 – 0.78	0.29	0.14 – 0.60	1.18	0.43 – 3.24	2.05	0.93 – 4.49	2.00	0.67 – 5.97
South	0.27	0.13 – 0.56	0.36	0.17 – 0.74	1.16	0.50 – 2.72	1.81	0.84 – 3.90	2.23	0.98 – 5.08
West	0.59	0.26 – 1.34	0.45	0.22 – 0.93	1.56	0.63 – 3.84	1.45	0.67 – 3.14	1.85	0.75 – 4.56
										
**MSA status**										
MSA	1.00	...	1.00	...	1.00	...	1.00	...	1.00	...
Non-MSA	0.45	0.21 – 0.96	0.77	0.38 – 1.53	1.96	1.06 – 3.63	0.90	0.40 – 2.07	2.90	0.64 – 13.08
										
**Chronicity**										
Acute (initial visit)	1.00	...	1.00	...	1.00	...	1.00	...	1.00	...
Chronic (follow-up visit)	0.87	0.48 – 1.59	1.16	0.72 – 1.87	1.54	0.87 – 2.75	0.56	0.37 – 0.86	0.35	0.19 – 0.64
										
**IPA etiology**										
No	1.00	...	1.00	...	1.00	...	1.00	...	1.00	...
Yes	2.34	1.49 – 3.68	2.38	1.50 – 3.77	0.74	0.44 – 1.27	1.19	0.83 – 1.73	0.62	0.36 – 1.08
										
**PCP status**										
Non-PCP or unknown	1.00	...	1.00	...	1.00	...	1.00	...	1.00	...
PCP	1.59	0.67 – 3.76	1.10	0.56 – 2.17	1.38	0.72 – 2.67	1.62	1.05 – 2.49	1.98	1.19 – 3.28
										
**Type of physician**										
Doctor of Medicine	1.00	...	1.00	...	1.00	...	1.00	...	1.00	...
Doctor of Osteopathy	1.53	0.80 – 2.92	1.02	0.46 – 2.26	0.70	0.31 – 1.58	0.43	0.24 – 0.76	0.44	0.18 – 1.11
										
**Physician specialty**										
Family (general) medicine	0.30	0.12 – 0.74	0.71	0.32 – 1.59	0.99	0.46 – 2.12	1.52	0.79 – 2.92	2.38	1.06 – 5.35
Internal medicine	0.15	0.05 – 0.40	0.42	0.13 – 1.32	0.63	0.25 – 1.62	0.68	0.28 – 1.69	0.73	0.28 – 1.87
Neurology	0.24	0.06 – 0.94	0.50	0.18 – 1.39	0.88	0.38 – 2.05	0.94	0.43 – 2.04	0.86	0.38 – 1.98
Orthopedics	1.55	0.59 – 4.05	1.01	0.40 – 2.55	0.81	0.33 – 2.00	0.97	0.39 – 2.40	0.45	0.16 – 1.31
All other specialties	1.00	...	1.00	...	1.00	...	1.00	...	1.00	...
										
**Shared physician care**										
No or unknown	1.00	...	1.00	...	1.00	...	1.00	...	1.00	...
Yes	1.13	0.62 – 2.08	1.08	0.62 – 1.87	1.61	0.95 – 2.76	1.28	0.70 – 2.36	1.02	0.60 – 1.75

Abbreviations: CI, confidence interval; IPA, injury, poisoning, or adverse effect of medical treatment; MSA, metropolitan statistical area; NSAID, nonsteroidal anti-inflammatory drug; OR, odds ratio; PCP, primary care physician.

*Only initial visits for acute problems or follow-up visits for chronic problems were included.

†Adjusted for all variables shown.

There was an association between age and drugs ordered (P = .01) during primary LBP patient visits (Table 5). Drugs were ordered less often in the young (<25 years) than in the referent age category (25–44 years). Drugs were ordered more often among non-Whites (P = .01) and by primary care physicians (P = .01), particularly family (general) medicine physicians. Similarly, primary care physicians were more likely to order NSAIDs (P = .03), although osteopathic physicians were less likely to order NSAIDs than allopathic physicians (P = .004). There was an association between age and narcotic analgesic use (P = .05), with less use in older patients (>65 years) compared with the referent age category (25–44 years). Narcotic analgesics were prescribed less often in Hispanics (P = .01) and more often in non-MSAs (P = .03).

## Conclusion

This study helps shed more light on LBP that motivates patients to seek medical care. More than 40% of LBP patient visits were provided by family (general) medicine physicians, comprising one of every 17 patient visits for this specialty. However, orthopedic surgeons provided a slightly greater percentage of LBP visits as part of their specialty case mix (1 of every 16 patient visits). Further, a substantial percentage of LBP patient visits (45%) were provided by non-primary care physicians. During 2003–2004 there were more than twice as many patient visits annually for LBP than reported in the 1990 NAMCS; however, the percentage of patient visits attributed to LBP (3%) and the percentage of LBP patient visits provided by primary care physicians (55%) remained remarkably similar to what was reported in the 1980s and early 1990s [20]. Non-primary care services generally are considered inappropriate for patients with *non-specific *LBP [10]. Thus, this study suggests that the recommended shift to primary care physicians for medical management of non-specific LBP has not occurred over the past two decades.

Injuries were the strongest risk factor associated with LBP patient visits (OR, 3.38; 95%, 2.75–4.14). There were also characteristic patterns of LBP patient visits according to age and geographic region. Patients other than those 25–44 years of age were less likely to seek medical care for LBP. Unavailable, and therefore uncontrolled, variables that could potentially explain the observed age distribution of LBP patient visits include occupational risk factors such as manual handling of materials, bending and twisting, whole-body vibration, and lifting for more than three-fourths of the work day [21]. Patient visits for LBP were less likely to occur in the Midwest and South than in the Northeast (OR, 0.56; 95% CI, 0.40–0.77 for each contrast). It is unclear if these geographic findings reflect the epidemiology of LBP in the United States or if they are confounded by other uncontrolled variables. While relatively little is known about risk factors in the transition from acute to chronic LBP, this study suggests that injuries are *not *associated with progression of LBP.

Osteopathic physicians were more likely than allopathic physicians to provide medical care during LBP patient visits (OR, 2.61; 95% CI, 1.75–3.92). The physician specialties most likely to provide LBP patient visits were family (general) medicine, internal medicine, neurology, and orthopedic surgery. These findings are consistent with previous studies [10,22]. There was an even stronger association between osteopathic physicians and chronic LBP patient visits (OR, 4.39; 95% CI, 2.47–7.80). However, physician specialists in family (general) medicine, internal medicine, and neurology were not more likely than other physician specialists to provide chronic LBP patient visits. These findings, coupled with the greater use of shared physician care in chronic LBP (OR, 2.11; 95% CI, 1.30–3.44), suggest that osteopathic physicians are often used to complement the conventional medical management of chronic LBP by providing spinal manipulation.

At least eleven national clinical guidelines for LBP medical management in the primary care setting were published between 1994 and 2000 [23]. An updated review of national clinical guidelines summarized recommendations according to LBP chronicity [24]. For acute LBP, radiographs were not considered useful for diagnosis of non-specific LBP. Recommended treatments included advising patients to remain active (although back-specific exercises were not considered effective), and ordering paracetamol or NSAIDs (muscle relaxants or narcotic analgesics may be considered as well). In contrast to acute LBP, few guidelines existed for the medical management of chronic LBP. Recently, however, European guidelines have been established for the management of chronic non-specific LBP [25]. These guidelines do not recommend radiographs or other diagnostic imaging tests unless a specific cause is strongly suspected. They recommend brief educational interventions (specifically including supervised exercise therapy), cognitive behavioral therapy, and short-term use of NSAIDs or weak narcotic analgesics for pain relief. They generally do not recommend physical therapies (although spinal manipulation may be considered) or surgery (unless all other recommended conservative treatments have been tried and failed over a period of at least two years).

This study suggests that cognitive behavioral therapy (as proxied by mental health counseling) may be under-utilized in the medical management of chronic LBP (6% of patient visits), which is often characterized by depression and somatization [26]. Nonsteroidal anti-inflammatory drugs were the most commonly used drugs for acute LBP (49% of patient visits); however, they were less likely to be used for chronic LBP (28% of patient visits). Non-narcotic analgesics were infrequently used for either acute or chronic LBP (6% of patient visits overall). The reported percentage of chronic back patients prescribed narcotic analgesics varies widely, from 3% to 66%, based on the treatment setting [27]. The present study found the relevant percentage to be 28%. Almost two million surgical procedures (about one million annually) were ordered, scheduled, or performed during primary LBP patient visits. Not surprisingly, surgical procedures were more frequently associated with chronic LBP patient visits compared with acute LBP patient visits (OR, 12.62; 95% CI, 3.18–50.07). Together, the findings of this study reinforce the caricature of LBP medical care in the United States as being overspecialized, overinvasive, and overexpensive [10].

The medical management of LBP varies substantially between practitioners and countries [24,28]. Differences were observed in this study with regard to type of physician provider, physician specialty, and geographic region. Osteopathic physicians were less likely than allopathic physicians to order NSAIDs for LBP (OR, 0.43; 95% CI, 0.24–0.76). This is consistent with the theory that osteopathic physicians are less likely to prescribe drugs for LBP because they may use spinal manipulation as an alternative to drugs [29]. Previous studies including an analysis of older NAMCS data [20] and a randomized controlled trial [30] have provided evidence to help support this theory. Family (general) medicine physicians were less likely to provide exercise counseling, but were more likely to order drugs for LBP. Time constraints during patient visits, particularly in a managed care environment, represent a possible explanation for the latter findings [31]. Patient counseling was less often provided outside the Northeast in this study. A strong predictor of patient counseling, including exercise counseling, was having had an injury as the reason for seeking medical care for LBP (OR, 2.38; 95% CI, 1.50–3.77).

Although this study involved a large, nationally representative sample of patient visits for ambulatory medical care in the United States, there are several limitations of this study that should be noted. The study involved the epidemiology and medical management of LBP that was of a magnitude sufficient to prompt patients to visit physician offices for ambulatory medical care. Further, the study was limited by the NAMCS patient record form to patient visits in which LBP was among the three most important reasons for seeking medical care. Thus, this may more properly be considered a study of the epidemiology and medical management of clinically significant LBP in the ambulatory medical care environment. Although the measurement of incidence or prevalence rates was not an objective of the study, all patient visits in which LBP was recorded as a reason for seeking medical care were included in the epidemiological analyses to capture the maximal number of incident or prevalent LBP cases and thereby to provide more precise statistical estimates. Nevertheless, similar results were observed in the epidemiological analyses when only primary LBP patient visits were included (Table 1). The medical management analyses, however, were limited to only primary LBP patient visits to avoid potential confounding by other more important reasons for seeking medical care.

Simplifying assumptions were made in certain analyses because of limitations inherent in the NAMCS patient record form. Patient visits attributed to back (RFV code 1905) and low back (RFV code 1910) symptoms were combined because there were no substantive differences in the characteristics associated with these reported reasons for seeking medical care (Table 1). All patient visits attributed to back symptoms were assumed to involve back pain, although 4% of such visits involved unspecified back symptoms and another 2% involved such other back symptoms as cramps, contractures, spasms, limitation of movement or stiffness, or weakness. Similarly, with regard to etiology, all of the 19.7 million patient visits in which an IPA was reported (using a dichotomous patient record form item) were assumed to involve an injury, although in the subset of 8.4 million patient visits in which the specific IPA was described, up to 11% may have involved iatrogenic, environmental, or other etiologies. The elements of LBP medical management were assessed with survey items that asked whether the relevant element was "ordered, scheduled, or performed." However, it was impossible to confirm whether the reported elements actually occurred within the relevant patient visit or were eventually performed by the reporting physician.

Several analyses yielded imprecise results because they were based on less than 30 NAMCS patient visits or because the SE was greater than 30% of the NPE. Most often this occurred with less common characteristics (internal medicine physicians) or elements of LBP medical management (weight reduction and mental health counseling, physiotherapy, and surgical procedures), or in stratified (subgroup) analyses. Thus, racial minority groups were combined in a "non-White" group to partially overcome this limitation. Hospital admission could not be studied as an element of LBP medical management because of the limited number of observations.

In conclusion, this study found that the percentage of LBP visits provided by primary care physicians in the United States remains suboptimal. Medical management of LBP, particularly chronic LBP, appears to over-utilize surgery relative to more conservative measures such as patient counseling, non-narcotic analgesics, and other drug therapies. Osteopathic physicians are more likely to provide LBP care, and less likely to use NSAIDs during such visits, than their allopathic counterparts. In general, LBP medical management does not appear to be in accord with evidence-based guidelines.

## Competing interests

JCL is Editor-in-Chief of *Osteopathic Medicine and Primary Care*. He was not involved in the review of the manuscript or in the editorial decision with regard to its suitability for publication.
